# External Validation of the Individualized Prediction of Breast Cancer Survival (IPBS) Model for Estimating Survival after Surgery for Patients with Breast Cancer in Northern Thailand

**DOI:** 10.3390/cancers14235726

**Published:** 2022-11-22

**Authors:** Thanapat Charumporn, Nutcha Jarupanich, Chanawin Rinthapon, Kantapit Meetham, Napat Pattayakornkul, Teerapant Taerujjirakul, Krittai Tanasombatkul, Chagkrit Ditsatham, Wilaiwan Chongruksut, Areerak Phanphaisarn, Donsuk Pongnikorn, Phichayut Phinyo

**Affiliations:** 1 Department of Family Medicine, Faculty of Medicine, Chiang Mai University, Chiang Mai 50200, Thailand; 2 Center for Clinical Epidemiology and Clinical Statistics, Faculty of Medicine, Chiang Mai University, Chiang Mai 50200, Thailand; 3 Division of Head, Neck, and Breast Surgery, Department of Surgery, Clinical Surgical Research Center, Faculty of Medicine, Chiang Mai University, Chiang Mai 50200, Thailand; 4 Division of Research, Department of Surgery and Clinical Surgical Research Center, Faculty of Medicine, Chiang Mai University, Chiang Mai 50200, Thailand; 5 Department of Orthopaedics, Faculty of Medicine, Chiang Mai University, Chiang Mai 50200, Thailand; 6 Vejjarak Lampang Hospital, Department of Medical Services, Ministry of Public Health, Lampang 52130, Thailand; 7 Musculoskeletal Science and Translational Research Cluster, Chiang Mai University, Chiang Mai 50200, Thailand

**Keywords:** breast neoplasms, adult, female, prognosis, statistical models

## Abstract

**Simple Summary:**

Recently, a prediction model was developed specifically for predicting the probability of survival and disease progression five years after diagnosis for Thai female patients with breast cancer. The model was composed of twelve routinely available clinical predictors. Even though it seemed to provide accurate predictions in the development dataset, it has never been tested in other external datasets. This study validated this new prediction model, entitled IPBS (Individualized Prediction of Breast cancer Survival), in another dataset of Northern Thai female patients with breast cancer and found that the model carried an acceptable discriminative ability comparable to when it was developed. Nonetheless, model recalibration to each specific context is encouraged, as it may overestimate the probability of events when the underlying baseline survival of the patient cohort is different from the development dataset.

**Abstract:**

The individualized prediction of breast cancer survival (IPBS) model was recently developed. Although the model showed acceptable performance during derivation, its external performance remained unknown. This study aimed to validate the IPBS model using the data of breast cancer patients in Northern Thailand. An external validation study was conducted based on female patients with breast cancer who underwent surgery at Maharaj Nakorn Chiang Mai hospital from 2005 to 2015. Data on IPBS predictors were collected. The endpoints were 5-year overall survival (OS) and disease-free survival (DFS). The model performance was evaluated in terms of discrimination and calibration. Missing data were handled with multiple imputation. Of all 3581 eligible patients, 1868 were included. The 5-year OS and DFS were 85.2% and 81.9%. The IPBS model showed acceptable discrimination: C-statistics 0.706 to 0.728 for OS and 0.675 to 0.689 for DFS at 5 years. However, the IPBS model minimally overestimated both OS and DFS predictions. These overestimations were corrected after model recalibration. In this external validation study, the IPBS model exhibited good discriminative ability. Although it may provide minimal overestimation, recalibrating the model to the local context is a practical solution to improve the model calibration.

## 1. Introduction

Breast cancer has recently become one of the most prevalent malignancies worldwide, with over 2 million new cases diagnosed in 2020 [1]. With continuous population growth, an improvement in accessibility to early mammographic screening, and a higher fraction of the population exposed to common risk factors, breast cancer incidence is projected to double the current figure within the year 2070 [2]. In Thailand, the age-standardized incidence rate of breast cancer has shown a steady rise from 18 per 100,000 women-years in 1998 to almost 40 per 100,000 women-years in 2020 [3]. This rising pattern of age-standardized incidence rates has been similarly observed in both developed and developing parts of the world [4].

However, there were still significant disparities in terms of breast cancer survival. From the recent Global Surveillance report, the five-year net survival was over 85% in the developed countries, whereas in the developing countries where healthcare resources (e.g., the number of healthcare personnel and access to effective therapy) are generally limited, and diagnosis is often delayed, the survival probability was much lower at only 65% [5]. As more than half of all patients are diagnosed within these less developed countries, breast cancer is certainly a significant burden to both individuals and public health [4].

During the past decades, the survival rates of patients with breast cancer have significantly improved, owing to both an advance in breast cancer therapy and an early screening strategy [6]. Nonetheless, the survival rates still varied largely across patients with different baseline prognoses [4]. Accurate prognostication of patient survival after a cancer diagnosis is paramount to clinicians and patients during shared decision-making about optimal treatment planning [7]. The development of multivariable clinical prediction models that provide absolute survival prediction for each individual is a current rising trend [8,9]. Over the years, several prognostic tools for breast cancer have been developed, validated, and implemented, such as the Nottingham prognostic index (NPI) [10], Adjuvant! [11], OPTIONS [12], and PREDICT [13]. Even though most of the tools have been proven to provide accurate predictions in many settings, one validation study from Thailand reported an underestimation of the predicted survival by some of these western-derived models and suggested that a prognostic model specific for Thai breast cancer patients be developed [14].

In 2021, the individualized prediction of breast cancer survival (IPBS) model was derived using the cohort data of breast cancer patients registered in the Network of National Cancer Institutes of Thailand [15]. The IPBS model is composed of twelve clinically-available predictors and is able to provide survival predictions with an acceptable discriminative ability and a good calibration. However, as no validation study has been conducted, the external performance of the IPBS remains unknown. This study aimed to externally validate the IPBS model in predicting the five-year overall survival (OS) and disease-free survival (DFS) using the cohort data of breast cancer patients who underwent surgical operations at Maharaj Nakorn Chiang Mai hospital.

## 2. Materials and Methods

### 2.1. Study Design

An external validation study of the IPBS model was conducted using a retrospective observational cohort design. Female patients with breast cancer registered in the Chiang Mai Cancer registry and underwent surgical treatment at Maharaj Nakorn Chiang Mai hospital from 1 January 2005 to 31 December 2015 were eligible for inclusion. The Institutional Review Board of the Faculty of Medicine, Chiang Mai University, approved the study protocol (FAM-2563–07779). In addition, we followed the TRIPOD (Transparent Reporting of a multivariable prediction model for Individual Prognosis Or Diagnosis) statement for reporting this study [9].

### 2.2. Study Patients

Patients who met the following criteria were included: (1) female patients aged between 18 and 90 years with primary invasive breast cancer based on histopathologic diagnosis, and (2) patients who underwent definite surgical treatment for breast cancer. We excluded patients who had one of the following criteria: (1) patients with stage IV breast cancer or metastatic breast cancer, (2) patients with a history of receiving neoadjuvant chemotherapy, pre-operative hormonal therapy, or radiotherapy, (3) patients with synchronous tumor, (4) patients with inflammatory breast cancer or Paget’s disease, and (5) patients with incomplete data, regarding the date of operation or national identification number. The investigators verified every included patient diagnosis and cross-checked it with the electronic medical database.

### 2.3. Data Collection

Patient clinical data (age at diagnosis, nationality, type of medical insurance, marital status, menopausal status), tumor factors (tumor size, location, clinical stage, histological type, tumor grade, number of nodes positive, number of examined lymph nodes, lymphovascular invasion (LVI), pathological stage, Ki-67 proliferation index, estrogen receptor (ER) status, progesterone receptor (PR) status, human epidermal growth factor receptor 2 (HER-2) status), and treatment factors (surgical procedure, adjuvant chemotherapy status, adjuvant targeted therapy (e.g., trastuzumab), adjuvant radiotherapy status, adjuvant hormonal therapy status) were retrieved from medical records. Tumor factor data were extracted from associated pathological reports. The ER or PR positivity was defined as 1% or more positive tumor cells with nuclear staining. The HER-2 positivity was defined as either a score of 2+ or 3+ by immunohistochemistry.

We also extracted and collected the baseline clinicopathologic characteristics data from the development study by Pongnikorn et al. [15] to assess the degree of relatedness between the two datasets.

### 2.4. The IPBS Model

The IPBS model consists of twelve practical predictors, including age at surgical treatment, menopausal status (premenopause or postmenopause), pathological staging (stage I, II, or III), tumor type (ductal carcinoma or others), histological grading (grade I, II, or III), tumor size in millimeters, lymphovascular invasion status (presence or absence), number of positive axillary lymph nodes (0, 1–3, or ≥4), ER (positive or negative), PR (positive or negative), HER-2 status (positive or negative), and type of surgical treatment (mastectomy or breast conserving surgery). According to the development dataset, the baseline 5-year OS and 5-year DFS were 0.893 and 0.889, respectively. The details on how to calculate the prognostic index of 5-year OS and 5-year DFS by the IPBS model are shown in Appendix A (Table A1).

### 2.5. Study Outcomes

The primary endpoints for prediction were 5-year OS and 5-year DFS. OS event was defined as death from any cause during follow-up, whereas DFS event was defined similarly to the IPBS development study, which was any invasive relapse (including ipsilateral recurrence), any appearance of a second primary cancer (including contralateral breast cancer), any appearance of distant metastasis, and death from any cause, whichever occurred first [16]. The survival time for both OS and DFS events started at the date of surgery. All patients included in the analysis were followed up until 5 years after their index date of surgery. Patients who had no event during the follow-up period were censored at 5 years.

### 2.6. Statistical Analyses

#### 2.6.1. Study Size Estimation

Our study size estimation was based on the previous guidance on sample size for designing an external validation study by Collins et al., which suggests that a minimum of 100 to 200 events are required for external validation of a prognostic model [17]. According to our preliminary review of patients diagnosed with breast cancer registered in the Chiang Mai Cancer Registry between 2005 and 2015, there were at least 820 deaths from 4096 patients. Provided that about half of these might be excluded, the remaining number of death events would still be sufficient.

#### 2.6.2. Handling of Missing Data

As we anticipated that there would be missing data on several prognostic variables of the IPBS model in our validation dataset, we used multiple imputation with chained equation (MICE) to replace the missing values. We followed a two-stage calculation using a quadratic rule to estimate the number of imputations required for MICE to achieve replicable parameters and their standard error estimates [18]. External validations were performed within each imputation dataset. The estimated parameters were then pooled with Rubin’s rules [19].

In this study, three analytic approaches for handling missing data were performed. The first analytic approach used multiple imputations that included the data on the observed outcomes as independent variables during MICE modeling. As the analysis concerned survival outcomes, Nelson–Aalen cumulative hazard estimates were used. The second approach was the use of multiple imputation that did not include the data on the cumulative hazard estimates. This approach would represent the performance of the model in real-life settings where the values of the observed outcomes were unknown [20]. The last approach was the complete-case analysis, where only patient records with complete data on IPBS predictors were included for analysis.

#### 2.6.3. Descriptive and Comparative Analysis

Statistical analyses were performed with Stata version 17 (StataCorp, College Station, TX, USA). Continuous data were summarized by mean and standard deviation or median and interquartile range (IQR) based on the underlying distribution. Frequency and percentage were used for the description of categorical data. Missing data was labeled and presented as an unknown dummy variable. Survival probability was estimated using Kaplan–Meier methods. Log-rank test was used to explore the association between each prognostic factor and OS.

The clinical characteristics and the association of prognostic factors for OS in the validation dataset were comparatively tabulated with those in the development dataset. The degree of relatedness was evaluated using the standardized difference (STD). Characteristics with an absolute STD value of more than 10% were considered significant differences between datasets [21].

#### 2.6.4. Evaluation of External Performance

The model performance was assessed in two aspects: discriminative ability and calibration. Harrell’s C-statistics was used to represent the model discrimination. Calibration was evaluated using calibration plots, expected-to-observed ratio (E:O ratio), and calibration slope. The expected-to-observed ratio of less than 1.0 indicated underestimation, whereas overestimation was suggested when these parameters were greater than 1.0.

For comparative purposes, the external performance of the updated version of PREDICT model (PREDICT v2) for predicting a 5-year OS was estimated [22]. However, as the specific model equation and baseline probability for DFS outcomes were not reported for the PREDICT v2, and we did not evaluate the performance in terms of DFS prediction. The details on how to calculate the prognostic index of 5-year OS by the PREDICT v2 model are shown in Appendix A (Table A2).

In case the IPBS or the PREDICT v2 model showed poor calibration in this validation dataset, model updating would be performed to improve the calibration of these prediction models in the validation set. In this study, model recalibration would be conducted by readjusting the model intercept or the baseline survival probability to that of our validation population while all other coefficients remained the same as originally proposed. This was achieved by refitting the Cox’s model in the validation dataset, while the linear predictors were included as an offset term [23]. This approach would allow us to correct calibration-in-the-large of the model, which is the common issue during external validation due to a mismatch in the overall observed event rate and the predicted risk [24].

An exploratory subgroup analysis of model performance was performed based on the histological subtypes of breast cancer and the pathological staging. The estimated subgroup C-statistics and calibration slope was based on a randomly selected sample of multiple imputed datasets, including the cumulative hazard of events.

## 3. Results

### 3.1. Patient Characteristics

**Figure 1:**
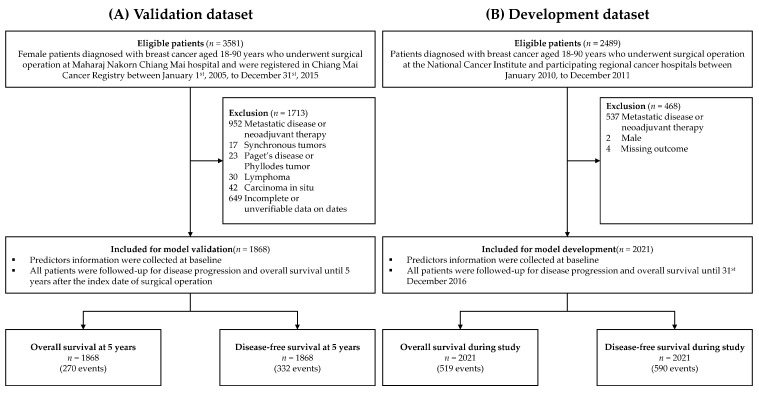
Patient flow diagrams of the validation and the development dataset by Pongnikorn et al. [15].

According to the Chiang Mai Cancer Registry, a total of 3581 female patients were diagnosed with breast cancer and underwent surgical operations at Maharaj Nakorn Chiang Mai hospital during the study period. Of these patients, 1713 were excluded from the analysis (Figure 1). The most common reasons for exclusion were patients with metastatic breast cancer and patients who received neoadjuvant chemotherapy. Finally, 1868 patients were included in the validation dataset. After a median follow-up of 5 years, there were 270 events for OS analysis and 332 events for DFS analysis. The 5-year OS and DFS of the patients in the validation dataset were 85.2% (95% confidence interval (CI) 83.5, 86.8%) and 81.9% (95% CI 80.1%, 83.6%), respectively.

In the validation dataset, the mean age at the time of surgery was 52.9 years, and around half of the patients were postmenopausal (49.6%). Regarding the characteristics of breast cancer, most of the patients were diagnosed with invasive ductal carcinoma (73.3%), with a pathological stage of I or II (54.2%) and a histological grade of II or III (80.6%). A larger proportion of patients had tumor size smaller than 30 mm (59.9%), without lymphovascular invasion (46.0%), absence of node involvements (50.9%), positive ER and PR status (57.1% and 49.8%), and negative HER-2 status (51.4%). The majority of the patients underwent surgical mastectomy (75.6%) and received adjuvant chemotherapy (77.3%). About half of the patients received hormonal therapy (56.6%) and adjuvant radiotherapy (54.5%). However, less than 10% of the patients were prescribed targeted therapy. The details on patient characteristics in both the validation and development datasets are shown in Table 1.

There were significant differences between the validation and the development dataset of the IPBS model in almost all of the presented features, except for the proportion of patients receiving radiotherapy (Table 1). The three clinicopathological characteristics with the highest standardized difference values were pathological staging (STD = 0.933), histological type (STD = 0.629), and histological grading (STD = 0.562).

### 3.2. Predictor–Outcome Associations

Both the validation and development datasets showed similar direction and statistical significance of predictor–outcome association patterns (Table 1), except for histological subtype, HER-2 status, type of surgery, and adjuvant chemotherapy. In the development dataset, there were no statistically significant differences in the 5-year OS between patients who were and were not prescribed adjuvant chemotherapy. In contrast, patients who did not receive chemotherapy were more likely to survive at 5-year in the validation dataset.

### 3.3. External Discrimination

For the discriminative ability, the median C-statistics of the IPBS model from multiple imputed datasets that include cumulative hazard of events were 0.728 (range 0.714–0.742) for 5-year OS and 0.689 (range 0.677–0.697) for 5-year DFS. For multiple imputation model that did not include cumulative hazard of events, the median and range of C-statistics of both 5-year OS and 5-year DFS were 0.706 (range 0.693–0.722) and 0.675 (range 0.663–0.685), respectively. In complete-case analysis, the discriminative ability of the IPBS model dropped below 0.7 for both 5-year OS (0.665, 95% CI 0.585, 0.745) and 5-year DFS (0.625, 95% CI 0.562, 0.689) (Table 2).

The median C-statistics of the PREDICT v2 model for predicting 5-year OS were 0.658 (range 0.638–0.672) and 0.644 (range 0.633–0.656) for multiple imputed datasets that included and did not include cumulative hazard of events, respectively. For complete-case analysis, the C-statistic of the PREDICT v2 model was estimated at 0.582 (95% CI 0.499, 0.664).

### 3.4. External Calibration

**Figure 2:**
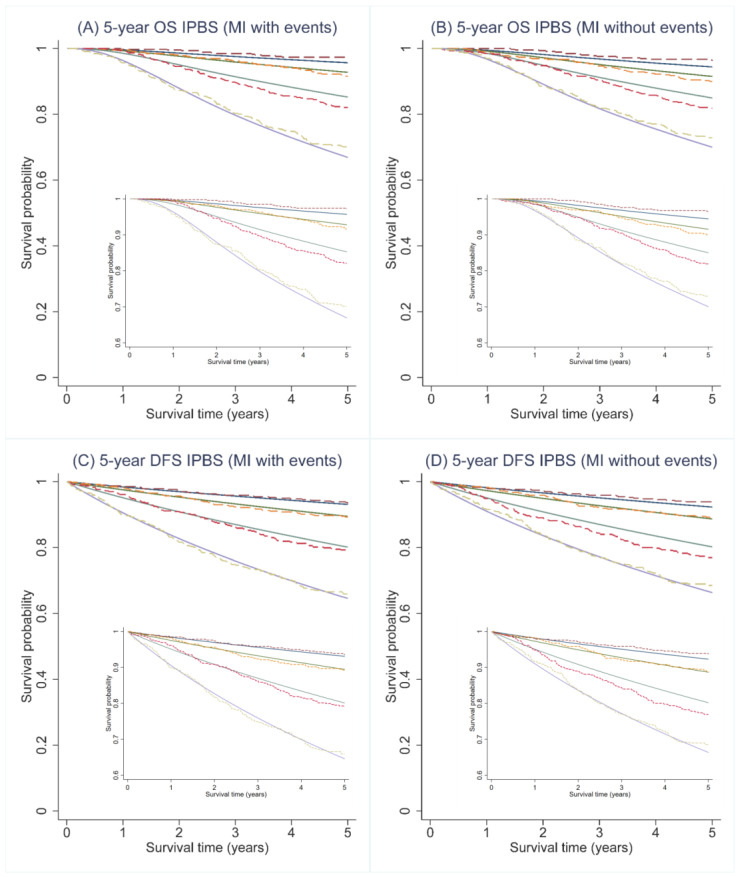
Predicted survival curves (solid lines) and the observed Kaplan–Meier estimates (dashed lines) for 5-year overall (OS) and disease-free survival (DFS) probability across 4 predicted risk groups using the IPBS model; (**A**,**C**) illustrate the results from multiple imputation methods, including cumulative hazard of events (MI with events), whereas (**B**,**D**) depicts the results from multiple imputation methods, not including cumulative hazard of events (MI without events).

**Figure A1:**
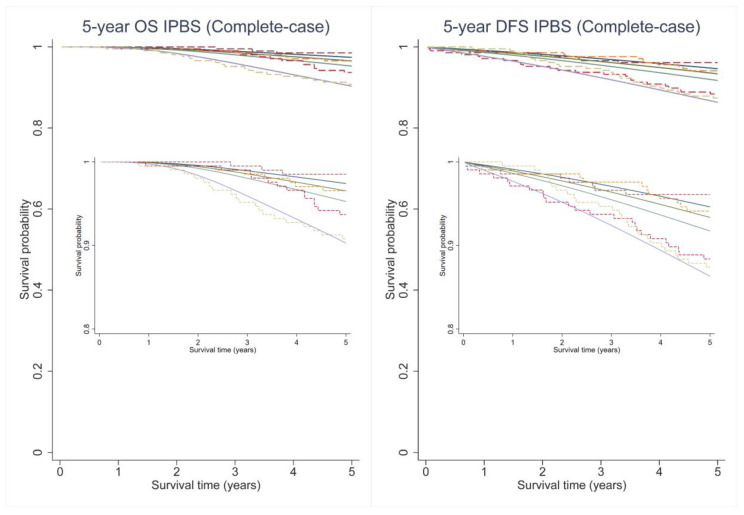
Predicted survival curves (solid lines) and the observed Kaplan–Meier estimates (dashed lines) for 5-year overall (OS) and disease-free survival (DFS) probability across 4 predicted risk groups using the IPBS model from complete-case analysis.

Regarding calibration, the IPBS model exhibited good agreement between the predicted survival probability and the observed proportion of 5-year OS and 5-year DFS for both multiple imputation approaches. Figure 2 visualizes the agreement between the predicted survival curves by the IPBS model and the observed Kaplan–Meier estimates across four risk quantiles for both MI approaches. For both 5-year OS and 5-year DFS, the IPBS model showed an apparent underestimation of the probability of events in the third risk quantile (Figure 2). The comparison of predicted survival curves and the observed Kaplan–Meier estimates for the complete-case analysis is shown in Appendix B (Figure A1).

According to the E:O ratio, the IPBS model modestly overestimated the 5-year risk of death by 4.6 to 5.2% while inversely underestimating the 5-year risk of disease progression by 10.7 to 11.2% for both MI approaches (Table 2). In the complete-case analysis, the IPBS model seriously overestimated the probability of death and disease progression by 2.47 and 1.54 times the proportions of the observed death and progression events, respectively (Table 2).

**Figure A2:**
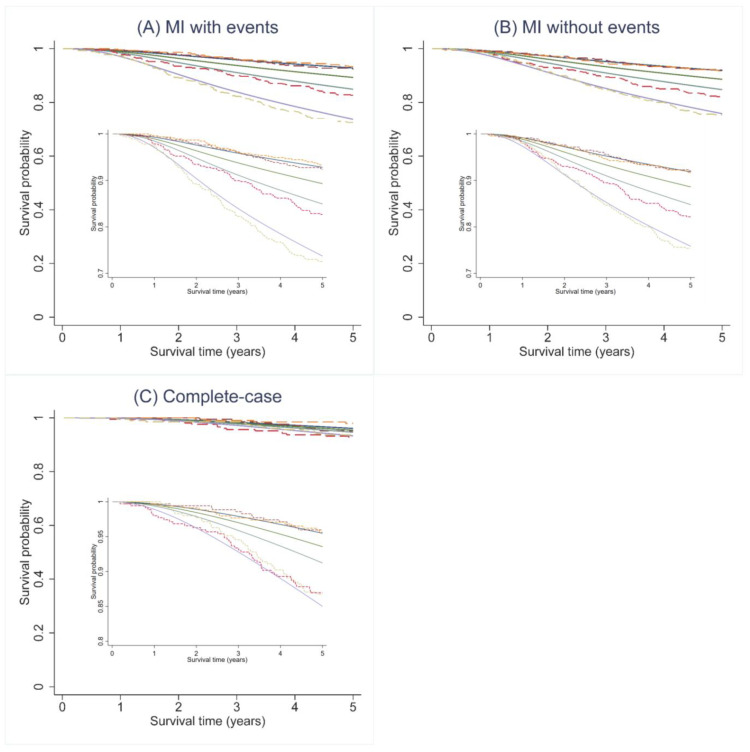
Predicted survival curves (solid lines) and the observed Kaplan–Meier estimates (dashed lines) for 5-year overall (OS) across 4 predicted risk groups using the PREDICT v2 model for all three analytic approaches. Figure (**A**) MI approach including cumulative hazard of events, (**B**) MI approach not including cumulative hazard of events, and (**C**) complete-case analysis.

According to the MI approaches, the PREDICT v2 model provided minimal underestimation of 5-year overall survival by 9.3 and 10.1% (Table 2). In contrast, the model’s predicted 5-year overall risk of mortality was significantly overestimated by almost two times the actual observed value in the complete-case analysis (Table 2). The figures visualizing the agreement between the observed and predicted survival curves across the 5-year follow-up period of the PREDICT v2 model for all three analytic approaches are provided in Appendix B (Figure A2).

### 3.5. Model Recalibration

**Figure 3:**
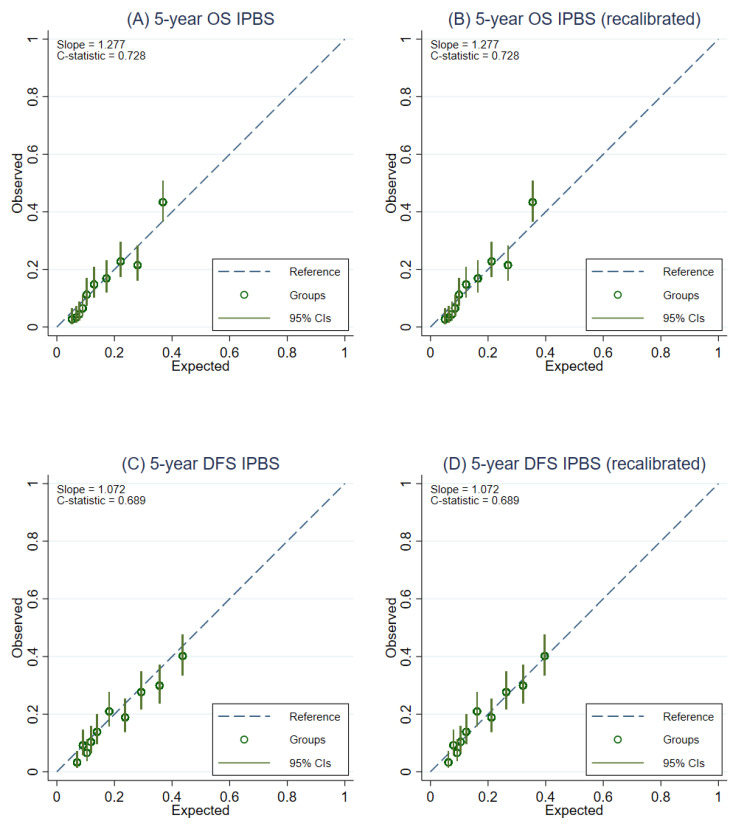
Calibration plots representing the external agreement between the observed and the expected probabilities of overall survival (OS) and disease-free survival (DFS) at 5 years of the IPBS model from multiple imputation methods, including cumulative hazard of events (MI with events). Figure (**A**) 5-year OS, (**B**) 5-year OS after model recalibration, (**C**) 5-year DFS, and (**D**) 5-year DFS after model recalibration.

**Figure 4:**
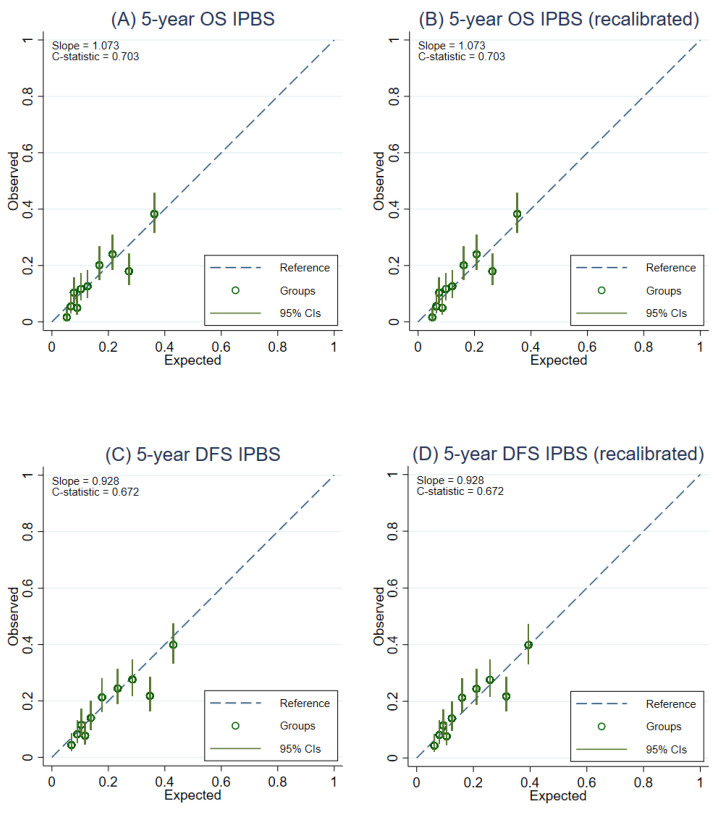
Calibration plots representing the external agreement between the observed and the expected probabilities of overall survival (OS) and disease-free survival (DFS) at 5 years of the IPBS model from multiple imputation methods not including cumulative hazard of events (MI without events). Figure (**A**) 5-year OS, (**B**) 5-year OS after model recalibration, (**C**) 5-year DFS, and (**D**) 5-year DFS after model recalibration.

**Figure A3:**
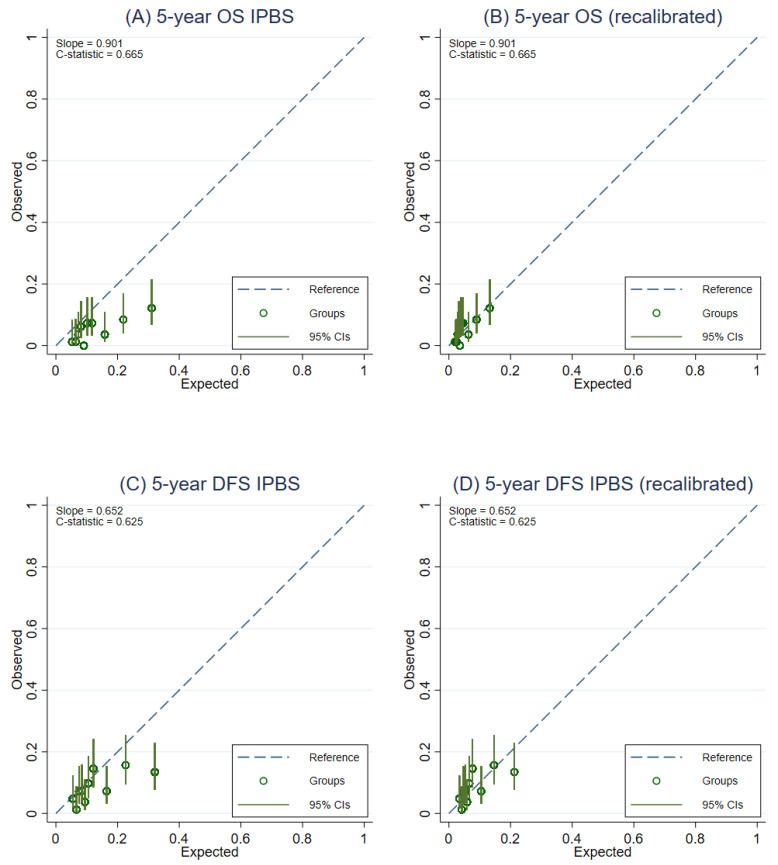
Calibration plots representing the external agreement between the observed and the expected probabilities of overall survival (OS) and disease-free survival (DFS) at 5 years of the IPBS model from complete-case analysis. Figure (**A**) 5-year OS, (**B**) 5-year OS after model recalibration, (**C**) 5-year DFS, and (**D**) 5-year DFS after model recalibration.

After recalibration of the IPBS model, the overall calibration of the IPBS model improved for both multiple imputation approaches and complete-case analysis (Table 2). Figure 3 compares the external calibration of the IPBS model for 5-year OS and 5-year DFS before and after model recalibration for the MI approach that includes cumulative hazard of events. In contrast, the comparison of the second MI approach is shown in Figure 4. The calibration plots of the IPBS model, before and after model recalibration from the complete-case analysis, are also presented in Appendix B (Figure A3).

**Figure A4:**
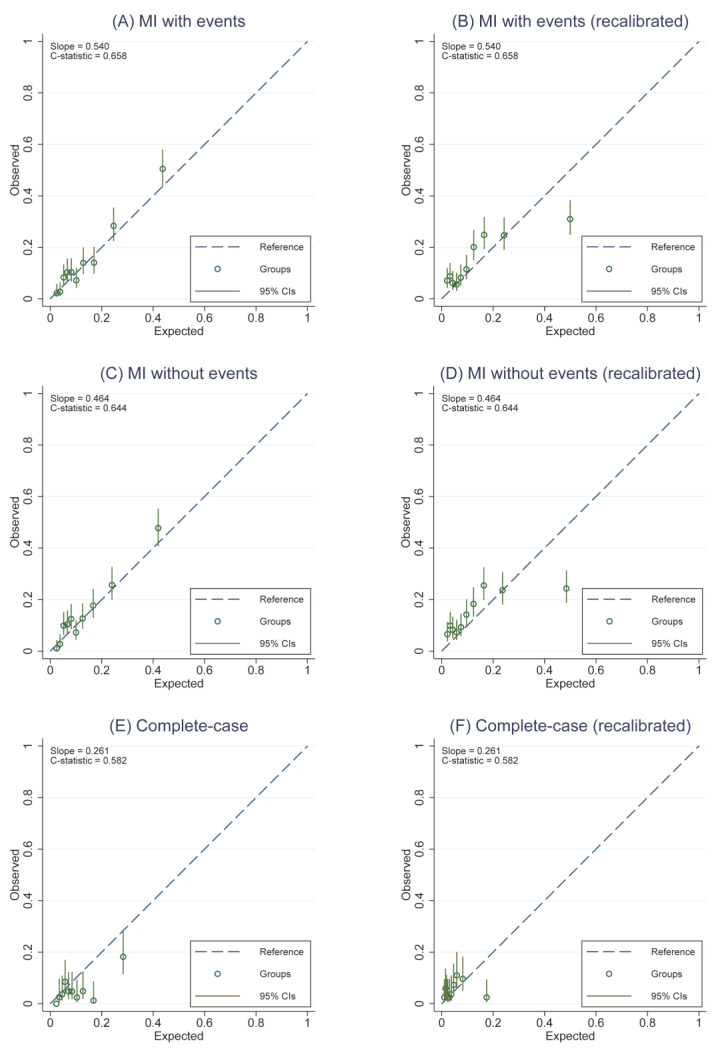
Calibration plots representing the external agreement between the observed and the expected probabilities of overall survival (OS) and disease-free survival (DFS) at 5 years of the PREDICT v2 for all three analytic approaches. Figure (**A**) 5-year OS from MI approach including cumulative hazard of events, (**B**) 5-year OS after model recalibration from MI approach including cumulative hazard of events, (**C**) 5-year OS from MI approach not including cumulative hazard of events, and (**D**) 5-year OS after model recalibration from MI approach not including cumulative hazard of events, (**E**) 5-year OS from complete-case analysis, (**F**) 5-year OS after model recalibration from complete-case analysis.

Improvements in model calibration were also observed for the PREDICT v2 model after recalibration, as observed through the changes in the E:O ratio toward 1.0. This was, however, more obvious in the complete-case analysis than in the MI approaches (Table 2). The calibration plots of the PREDICT v2 model (before and after recalibrating the baseline survival probability) are presented in Appendix B (Figure A4).

### 3.6. Exploratory Subgroup Analysis of Model Performance

The C-statistics and the calibration slope of the IPBS and PREDICT v2 model for predicting 5-year survival outcomes stratified by histological subtypes and pathological stage are shown in Appendix A (Table A3). It was observed that the performance of IPBS did not vary by the histological subtype of breast cancer. However, the IPBS model tended to show poor discrimination in patients with higher pathological stages. The PREDICT v2 model’s performance was affected by the histological subtype and pathological stage.

## 4. Discussion

To our knowledge, this study is the first to externally validate the performance of the IPBS model after its introduction in 2021. The IPBS model showed acceptable discriminative ability for predicting both outcomes in the validation dataset (median C-statistics 0.706 to 0.728 for 5-year OS and 0.675 to 0.689 for 5-year DFS). Based on our findings, the external discriminative ability of the IPBS model was somewhat similar to that of the development study (C-statistics 0.72 for OS prediction and 0.70 for DFS prediction) [15]. However, in terms of external calibration, IPBS minimally overestimated the probability of mortality and progression events (4.6 to 5.2% for 5-year OS and 10.7 to 11.2% for 5-year DFS). The external performance of the PREDICT v2 model in predicting 5-year OS was inferior to that of the IPBS model in terms of discriminative ability (median C-statistics 0.644 to 0.658 for 5-year OS).

To evaluate whether the results of our study represent the reproducibility or the transportability of the IPBS model, we assessed the relatedness between the validation and the development dataset [25]. Compared to the development data, our validation data showed significant differences in most of the clinicopathologic characteristics at diagnosis, such as the pathological staging and histologic grading. This heterogeneity in prognostic characteristics between the two datasets could also explain the differences in outcome occurrence and survival rates. The 5-year OS and 5-year DFS were obviously higher in the validation dataset than in the development dataset (OS 85.2% vs. 77.9% and DFS 81.9% vs. 74.0%) [15]. In spite of these differences, the direction of predictor–outcome associations was generally the same for both datasets. Overall, it might be concluded that the validation study was only partly related to the development study and that the results of this validation study would reflect the transportability of the IPBS model over reproducibility.

Despite the unrelatedness between the two datasets, the IPBS model still showed an acceptably robust performance in providing survival prediction for surgically-treated breast cancer patients. Nonetheless, there was still evidence that the IPBS model may provide a modest overestimation of mortality and disease progression events, which could be explained by the lower baseline survival probability in the development dataset [8]. When the IPBS model was recalibrated in the validation data, the overestimation of event rates disappeared. This important finding suggests that, while the discriminative ability of the IPBS model is well-preserved and that the derivation of new prognostic models or addition of other important features might not be necessary, the baseline survival probability should be recalibrated or tailored for each specific group of patients to achieve accurate predictions [26].

The observed superiority further strengthened the potential clinical applicability of the IPBS model for the Thai population in the external discriminative performance compared to the recently proposed PREDICT v2 model in our dataset. One previous study was conducted to validate the performance of the PREDICT v2 model in Thai patients with breast cancer in 2020 [14]. It was found that PREDICT v2 underestimated the overall survival probability, which was concordant with our findings. However, it was unclear whether recalibrating the model would correct this issue. The discriminative ability of the PREDICT v2 model was estimated at C-statistics 0.78 for 5-year OS, which was modestly higher than that of our study. This discrepancy could be explained by differences in study populations and the types of concordance statistics used for validation.

Our study carries both strengths and limitations. The main strength was the inclusion of a sufficiently large number of OS and DFS events that would be adequate for validating a prognostic model. Another important point was the use of real-world routinely collected data as the data source, which improved the generalizability of the validation results [27]. However, the use of real-world data also led to several issues that threatened the validity of our findings. First, less than half of the included patients had complete data on all twelve IPBS predictors. As a complete-case analysis may be subjected to a serious risk of selection bias, we employed two MICE approaches to handle the missing data in the analyses. We also performed and reported the performance measure from a complete-case analysis. Even though the results of the complete-case analysis were different from those of the MICE approaches, we believe this finding had no strong clinical implication, as a growing body of evidence has found that complete-case analysis often leads to errors, result misinterpretations, and impaired generalizability [28]. Second, a large proportion of eligible patients were excluded due to incomplete or unverifiable data on relevant dates. Finally, this validation study was based only on the data of breast cancer patients who were diagnosed and treated at a single tertiary care center in Northern Thailand. Although our results should be considered transportable to other distant populations, according to the unrelatedness of the datasets, further broader external validation studies are still encouraged. In addition, as the data on how other well-known western-derived prediction models perform in the Thai population is still limited, an independent validation study of these models would be of value to clinical practice.

## 5. Conclusions

The IPBS was externally validated using the cohort data of female breast cancer patients in one tertiary care center in Northern Thailand. The validation dataset was proven to be unrelated to the development dataset. Hence, the validation results should be considered in terms of transportability rather than reproducibility. The model was able to provide both OS and DFS predictions at 5 years after diagnosis with an acceptable discriminative ability comparable to when it was developed. However, it was apparent from our results that the model provided a minimal overestimation of event probability for both OS and DFS. Thus, recalibrating the IPBS model to the local context is suggested before clinical implementation.

## Figures and Tables

**Table 1:** Comparison of demographic and Clinicopathologic characteristics of the study patients between the validation and the development dataset by Pongnikorn et al. [15].

Characteristics	Validation Dataset				Development Dataset				STD
	Total (*n* = 1868)	5-Year OS		*p*-Value	Total (*n* = 2021)	5-Year OS		*p*-Value	
	*n* (%)	(%)	95% CI		*n* (%)	(%)	95% CI		
Age at surgery (year, mean ± SD)	52.9 ± 11.0				50.4 ± 10.6				
<50	768 (41.1)	88.0	85.5–90.1	0.004	1020 (50.5)	80.5	NR	0.001	0.189
≥50	1100 (58.9)	65.0	59.1–70.9		1001 (49.5)	75.2	NR		
Menopausal status									
Premenopause	619 (33.1)	88.7	85.9–91.0	<0.001	903 (44.7)	80.2	NR	0.012	0.401
Postmenopause	927 (49.6)	82.2	79.5–84.5		996 (49.3)	75.4	NR		
Unknown	322 (17.2)				112 (6.0)				
Pathological stage									
I	431 (23.1)	97.4	95.4–98.6	<0.001	286 (14.2)	91.3	NR	<0.001	0.933
II	580 (31.1)	93.1	90.6–94.9		979 (48.4)	84.9	NR		
III	366 (19.6)	88.5	84.7–91.4		744 (26.8)	63.8	NR		
Unknown	491 (26.3)				12 (0.6)				
Histological type									
Ductal	1370 (73.3)	89.0	87.2–90.6	0.011	1915 (94.8)	77.7	NR	0.140	0.629
Other types	376 (20.1)	93.5	90.5–95.6		106 (5.2)	82.1	NR		
Unknown	122 (6.5)								
Histological grade									
I	76 (4.1)	94.5	86.1–97.9	0.005	277 (13.7)	85.2	NR	<0.001	0.562
II	866 (46.4)	91.7	89.6–93.4		955 (47.2)	80.0	NR		
III	638 (34.2)	87.1	84.2–89.5		652 (47.2)	72.2	NR		
Unknown	288 (15.4)				53 (2.6)				
Tumor size (mm)									
<30	1119 (59.9)	91.0	89.1–92.5	<0.001	1014 (50.2)	82.6	NR	<0.001	0.349
≥30	649 (34.7)	77.5	74.0–80.5		1205 (59.6)	73.6	NR		
Unknown	100 (5.4)				132 (6.5)				
LVI									
Yes	739 (39.6)	83.2	80.3–85.7	<0.001	684 (33.9)	70.0	NR	<0.001	0.325
No	860 (46.0)	92.6	90.6–94.2		1205 (59.6)	82.0	NR		
Unknown	269 (14.4)				132 (6.5)				
Node									
0	950 (50.9)	93.1	91.3–94.6	<0.001	838 (41.5)	88.5	NR	<0.001	0.505
1–3	433 (23.2)	85.7	82.0–88.7		524 (25.9)	80.3	NR		
≥4	351 (18.8)	68.3	63.1–72.9		659 (32.6)	62.4	NR		
Unknown	134 (7.2)								
ER									
Positive	1067 (57.1)	90.5	88.5–92.1	<0.001	1237 (61.2)	82.7	NR	<0.001	0.174
Negative	670 (35.9)	80.1	76.9–83.0		718 (35.5)	69.6	NR		
Unknown	131 (7.01)				66 (3.3)				
PR									
Positive	930 (49.8)	91.5	89.5–93.1	<0.001	1026 (50.8)	84.1	NR	<0.001	0.165
Negative	805 (43.1)	80.7	77.8–83.3		925 (45.8)	71.1	NR		
Unknown	133 (7.1)				70 (3.4)				
HER-2 status									
Positive	760 (40.7)	86.0	83.4–88.3	0.417	687 (34.0)	74.5	NR	0.001	0.158
Negative	960 (51.4)	87.4	85.1–89.4		1110 (54.9)	80.6	NR		
Unknown	148 (7.9)				224 (11.1)				
Type of surgery									
Mastectomy	1412 (75.6)	89.2	87.4–90.7	0.076	1758 (87.0)	76.3	NR	<0.001	0.442
BCS	304 (16.3)	92.5	88.9–95.0		263 (13.0)	88.2	NR		
Unknown	152 (8.1)								
Chemotherapy									
Yes	1443 (77.3)	84.4	82.4–86.1	0.041	1696 (83.9)	78.1	NR	0.685	0.246
No	385 (20.6)	88.7	85.0–91.5		325 (16.1)	76.9	NR		
Unknown	40 (2.1)								
Hormonal therapy									
Yes	1058 (56.6)	90.2	88.2–91.8	<0.001	1053 (52.1)	85.5	NR	<0.001	0.137
No	717 (38.4)	78.4	75.2–81.2		900 (44.5)	69.8	NR		
Unknown	93 (5.0)				69 (3.4)				
Targeted therapy									
Yes	131 (7.0)	85.4	78.1–90.4	0.929	NR	NR	NR	NR	-
No	1670 (89.4)	85.3	83.5–87.0		NR	NR	NR		
Unknown	67 (3.6)								
RT									
Yes	1018 (54.5)	81.9	79.4–84.2	<0.001	1126 (55.7)	75.1	NR	<0.001	0.078
No	802 (42.9)	89.7	87.3–91.6		819 (40.5)	82.3	NR		
Unknown	48 (2.6)				76 (3.8)				

Abbreviations: CI, confidence interval; ER, estrogen receptor; HER-2, human epidermal growth factor receptor 2; LVI, lymphovascular invasion; OS, Overall survival; PR, progesterone receptor; RT, radiotherapy and BCS, breast conserving surgery; NR, Not reported; STD, standardized difference.

**Table 2:** External validation of 5-year overall and disease-free survival by the IPBS model in the validation dataset compared to the development dataset by Pongnikorn et al. [15] The performance of the PREDICT v2 model for predicting 5-year overall survival is also presented.

	Expected: Observed (E:O) Ratio	Calibration Slope	C-Statistics from Validation Dataset (Median, Range *)	C-Statistics from Development Dataset
Multiple imputations including cumulative hazard of events (n = 1868)				
5-year OS				
IPBS	1.052	1.277	0.728 (0.714–0.742)	0.72
Recalibrated IPBS	1.009	1.277	0.728 (0.714–0.742)	
PREDICT	0.907	0.540	0.658 (0.638–0.672)	
Recalibrated PREDICT	0.901	0.540	0.658 (0.638–0.672)	
5-year DFS				
IPBS	1.112	1.072	0.689 (0.677–0.697)	0.70
Recalibrated IPBS	0.996	1.072	0.689 (0.677–0.697)	
Multiple imputations not including cumulative hazard of events (n = 1868)				
5-year OS				
IPBS	1.046	1.073	0.706 (0.693–0.722)	0.72
Recalibrated IPBS	1.009	1.073	0.706 (0.693–0.722)	
PREDICT	0.899	0.464	0.644 (0.633–0.656)	
Recalibrated PREDICT	0.912	0.464	0.644 (0.633–0.656)	
5-year DFS				
IPBS	1.107	0.928	0.675 (0.663–0.685)	0.70
Recalibrated IPBS	1.003	0.928	0.675 (0.663–0.685)	
Excluding patients with incomplete data on predictors (complete-case analysis) (n = 837)				
5-year OS				
IPBS	2.471	0.901	0.665 (0.585, 0.745) ^†^	0.72
Recalibrated IPBS	0.999	0.901	0.665 (0.585, 0.745) ^†^	
PREDICT	1.954	0.261	0.582 (0.499, 0.664) ^†^	
Recalibrated PREDICT	0.967	0.261	0.582 (0.499, 0.664) ^†^	
5-year DFS				
IPBS	1.541	0.652	0.625 (0.562, 0.689) ^†^	0.70
Recalibrated IPBS	0.987	0.652	0.625 (0.562, 0.689) ^†^	

Abbreviations: IPBS, the Individualized Prediction of Breast Cancer Survival model; DFS, disease-free survival; OS, overall survival. * Median and range of C-statistics from multiple imputed datasets. ^†^ 95% confidence interval.

## Data Availability

The datasets used and analyzed during the current study are available from the corresponding author on reasonable request. The data are not publicly available, due to their containing information that could compromise the privacy of research patients.

## Appendix A

**Table A1:** List of IPBS model predictors and the equation used for estimating the survival probability of patients with breast cancer.

Variable	Predictors	Detail	Input Value
AGE	Age at surgery (year)		AGE = age at surgery–50
MENO	Menopausal status	Pre-menopause	MENO = 0
		Post-menopause	MENO = 1
SURG	Type of surgery	Mastectomy	SURG = 0
		Breast conserving surgery (BCS)	SURG = 1
STG	Pathological stage		
		I	STG2 = 0, STG3 = 0
		II	STG2 = 1, STG3 = 0
		III	STG2 = 0, STG3 = 1
HIST	Histological type	Others,	HIST = 0
		Ductal	HIST = 1
GRD	Histological grade		
		I	GRD2 = 0, GRD3 = 0
		II	GRD2 = 1, GRD3 = 0
		III	GRD2 = 0, GRD3 = 1
SIZE	Tumor size (mm)		SIZE = tumor size—30
LVI	Lympho-vascular invasion	No,	LVI = 0
		Yes	LVI = 1
NODE	Number of positive axillary lymph nodes (node)		
		0	NODE2 = 0, NODE3 = 0
		1–3	NODE2 = 1, NODE3 = 0
		≥4	NODE2 = 0, NODE3 = 1
ER	Estrogen receptor status	Negative	ER = 0
		Positive	ER = 1
PR	Progesterone receptor status	Negative	PR = 0
		Positive	PR = 1
HER2	HER-2 status	Negative	HER2 = 0
		Positive	HER2 = 1
CHEM	Chemotherapy		CHEM = 0.839
HORM	Hormonal therapy		HORM = 0.539
RADI	Radiotherapy		RADI = 0.579
OS_5_	Baseline 5-year overall survival probability		0.893
DFS_5_	Baseline 5-year disease-free survival probability		0.889
PI_OS_	Prognostic index of OS	0.0001 × AGE + 0.1681 × MENO − 0.2428×SURG + 0.0398 × STG2 + 0.5962 × STG3 + 0.4004 × HIST + 0.0021 × SIZE + 0.4655 × NODE2 + 0.8066 × NODE3 + 0.2071 × LVI + 0.1737 × GRD2 + 0.3126 × GRD3 − 0.1122 × ER − 0.1037 × PR + 0.0714 × HER2 − 0.4421 × CHEM − 0.5539 × HORM − 0.0246 × RADI	
PI_DFS_	Prognostic index of DFS	0.0018 × AGE + 0.1510 × MENO − 0.1956 × SURG + 0.2018 × STG2 + 0.6774 × STG3 + 0.3416 × HIST + 0.0016 × SIZE + 0.5409 × NODE2 + 0.7573 × NODE3 + 0.2435 × LVI + 0.3422 × GRD2 + 0.4226 × GRD3 − 0.1073 × ER − 0.0358×PR + 0.0973 × HER2 − 0.5737 × CHEM − 0.4825×HORM + 0.0050×RADI	
	Overall survival probability at 5-year	OS_5_^exp(PI^_OS_^)^	
	Disease-free survival probability at 5 years	DFS_5_^exp(PI^_DFS_^)^	

**Table A2:** List of PREDICT v2 model predictors and the equation used for estimating the survival probability of patients with breast cancer.

Variable	Predictors	Input value	Log HR
Model for breast cancer specific mortality for ER-negative breast cancer patients			
AGE_ERneg	Age at surgery (year)	Age–56.325	0.00894
SIZE_ERneg	Tumor size (mm)	(Tumor size /100)^1/2^ − 0.5090	2.109
NODES_ERneg	Number of positive axillary lymph nodes (node)	1/[(Number of nodes + 1)/10]^1/2^ − 1.72	−0.705
GRADE_ERneg	Histological grade	Histological grade (1,2,3)	0.259
PI_OS for ER-_	Prognostic index of OS for ER-negative patients	0.00894 × AGE_ERneg + 2.109 × (SIZE_ERneg) + (−0.705 × NODES_ERneg) + 0.259 × GRADE_ERneg	
Model for breast cancer specific mortality for ER-positive breast cancer patients			
AGE_Erpos1	Age at surgery (year)	(Age /10)^−2^ − 0.0287	34.53
AGE_Erpos2	Age at surgery (year)	(Age /10)^−2^ × ln(Age/10) − 0.0510	−34.20
SIZE_Erpos	Tumor size (mm)	ln(Tumor size /100) + 1.5452	0.7531
NODES_Erpos	Number of positive axillary lymph nodes (node)	ln((Nodes + 1)/10) + 1.3876	0.7069
GRADE_Erpos	Histological grade	Histological grade (1,2,3)	0.7467
SCREEN	Screen-detected	Not screen detected (0)	−0.2763
PI_OS for ER+_	Prognostic index of OS for ER-positive patients	(34.53 × AGE_Erpos1) + (−34.20 × AGE_ERpos2) + 0.7531 × SIZE_ERpos + 0.7069 × NODES_ERpos + 0.7467×GRADE_ERpos + (−0.2763 × 0)	
Model for non-specific mortality			
AGE_nonbreast	Age at surgery (year)	(Age/10)^2^ − 34.234	0.0698
PI_OS for non-breast_	Prognostic index for non-specific mortality	AGE_nonbreast × 0.0698	
BS_5_	Breast cancer specific mortality at 5 years	ER-negative patients	0.9805221
		ER-positive patients	0.8531408
NBS_5_	Non-breast cancer specific mortality at 5 years		0.9726993
	Overall survival probability at 5-year	ER-negative patients	BS_5_^exp(PIOS for ER-)^ × NBS_5_^exp(PIOS for non-breast)^
		ER-positive patients	BS_5_^exp(PIOS for ER+)^ × NBS_5_^exp(PIOS for non-breast)^

**Table A3:** Discriminative ability and calibration slope of the IPBS model by subgroups (histological subtypes, pathological stages, and estrogen receptor status) in a randomly selected sample of multiple imputed datasets, including cumulative hazard of events.

	IPBS Model				PREDICT v2	
	5-Year OS		5-Year DFS		5-Year OS	
	C-Statistics	Calibration Slope	C-Statistics	Calibration Slope	C-Statistics	Calibration Slope
Overall	0.728 (0.714–0.742) *	1.052	0.689 (0.677–0.697) *	1.112	0.658 (0.638–0.672)	0.540
Histological subtype						
Others	0.733	1.300	0.693	1.000	0.709	0.727
Ductal	0.735	1.321	0.695	1.124	0.659	0.494
Pathological stage						
I	0.748	2.465	0.712	2.026	0.648	0.801
II	0.696	1.570	0.656	1.268	0.618	0.440
III	0.658	1.486	0.642	1.416	0.567	0.240

Abbreviations: IPBS, the Individualized Prediction of Breast Cancer Survival model; DFS, disease-free survival; OS, overall survival. * Median and range of C-statistics from multiple imputed datasets.

## References

[1] Lei S., Zheng R., Zhang S., Wang S., Chen R., Sun K., Zeng H., Zhou J., Wei W. (2021). Global Patterns of Breast Cancer Incidence and Mortality: A Population-based Cancer Registry Data Analysis from 2000 to 2020. Cancer Commun..

[2] Soerjomataram I., Bray F. (2021). Planning for Tomorrow: Global Cancer Incidence and the Role of Prevention 2020–2070. Nat. Rev. Clin. Oncol..

[3] Bray F., Ferlay J., Laversanne M., Brewster D.H., Gombe Mbalawa C., Kohler B., Piñeros M., Steliarova-Foucher E., Swaminathan R., Antoni S. (2015). Cancer Incidence in Five Continents: Inclusion Criteria, Highlights from Volume X and the Global Status of Cancer Registration. Int. J. Cancer.

[4] Wilkinson L., Gathani T. (2022). Understanding Breast Cancer as a Global Health Concern. Br. J. Radiol..

[5] Allemani C., Matsuda T., Di Carlo V., Harewood R., Matz M., Nikšić M., Bonaventure A., Valkov M., Johnson C.J., Estève J. (2018). Global Surveillance of Trends in Cancer Survival 2000–14 (CONCORD-3): Analysis of Individual Records for 37,513,025 Patients Diagnosed with One of 18 Cancers from 322 Population-Based Registries in 71 Countries. Lancet Lond. Engl..

[6] Guo F., Kuo Y., Shih Y.C.T., Giordano S.H., Berenson A.B. (2018). Trends in Breast Cancer Mortality by Stage at Diagnosis among US Young Women. Cancer.

[7] Altman D.G. (2009). Prognostic Models: A Methodological Framework and Review of Models for Breast Cancer. Cancer Investing..

[8] Steyerberg E. (2009). Clinical Prediction Models: A Practical Approach to Development, Validation, and Updating. Statistics for Biology and Health.

[9] Collins G.S., Reitsma J.B., Altman D.G., Moons K.G. (2015). Transparent Reporting of a Multivariable Prediction Model for Individual Prognosis or Diagnosis (TRIPOD): The TRIPOD Statement. BMC Med..

[10] Haybittle J.L., Blamey R.W., Elston C.W., Johnson J., Doyle P.J., Campbell F.C., Nicholson R.I., Griffiths K. (1982). A Prognostic Index in Primary Breast Cancer. Br. J. Cancer.

[11] Ravdin P.M., Siminoff L.A., Davis G.J., Mercer M.B., Hewlett J., Gerson N., Parker H.L. (2001). Computer Program to Assist in Making Decisions about Adjuvant Therapy for Women with Early Breast Cancer. J. Clin. Oncol. Off. J. Am. Soc. Clin. Oncol..

[12] Campbell H.E., Gray A.M., Harris A.L., Briggs A.H., Taylor M.A. (2010). Estimation and External Validation of a New Prognostic Model for Predicting Recurrence-Free Survival for Early Breast Cancer Patients in the UK. Br. J. Cancer.

[13] Wishart G.C., Bajdik C.D., Azzato E.M., Dicks E., Greenberg D.C., Rashbass J., Caldas C., Pharoah P.D.P. (2011). A Population-Based Validation of the Prognostic Model PREDICT for Early Breast Cancer. Eur. J. Surg. Oncol. J. Eur. Soc. Surg. Oncol. Br. Assoc. Surg. Oncol..

[14] Polchai N., Sa-nguanraksa D., Numprasit W., Thumrongtaradol T., O-charoenrat E., O-charoenrat P. (2020). A Comparison Between the Online Prediction Models CancerMath and PREDICT as Prognostic Tools in Thai Breast Cancer Patients. Cancer Manag. Res..

[15] Pongnikorn D., Phinyo P., Patumanond J., Daoprasert K., Phothong P., Siribumrungwong B. (2021). Individualized Prediction of Breast Cancer Survival Using Flexible Parametric Survival Modeling: Analysis of a Hospital-Based National Clinical Cancer Registry. Cancers.

[16] Gourgou-Bourgade S., Cameron D., Poortmans P., Asselain B., Azria D., Cardoso F., A’Hern R., Bliss J., Bogaerts J., Bonnefoi H. (2015). Guidelines for Time-to-Event End Point Definitions in Breast Cancer Trials: Results of the DATECAN Initiative (Definition for the Assessment of Time-to-Event Endpoints in CANcer Trials)†. Ann. Oncol. Off. J. Eur. Soc. Med. Oncol..

[17] Collins G.S., Ogundimu E.O., Altman D.G. (2016). Sample Size Considerations for the External Validation of a Multivariable Prognostic Model: A Resampling Study. Stat. Med..

[18] von Hippel P.T. (2020). How Many Imputations Do You Need? A Two-Stage Calculation Using a Quadratic Rule. Sociol. Methods Res..

[19] Marshall A., Altman D.G., Holder R.L., Royston P. (2009). Combining Estimates of Interest in Prognostic Modelling Studies after Multiple Imputation: Current Practice and Guidelines. BMC Med. Res. Methodol..

[20] Hoogland J., van Barreveld M., Debray T.P.A., Reitsma J.B., Verstraelen T.E., Dijkgraaf M.G.W., Zwinderman A.H. (2020). Handling Missing Predictor Values When Validating and Applying a Prediction Model to New Patients. Stat. Med..

[21] Austin P.C. (2009). Balance Diagnostics for Comparing the Distribution of Baseline Covariates between Treatment Groups in Propensity-Score Matched Samples. Stat. Med..

[22] Candido dos Reis F.J., Wishart G.C., Dicks E.M., Greenberg D., Rashbass J., Schmidt M.K., van den Broek A.J., Ellis I.O., Green A., Rakha E. (2017). An Updated PREDICT Breast Cancer Prognostication and Treatment Benefit Prediction Model with Independent Validation. Breast Cancer Res..

[23] Booth S., Riley R.D., Ensor J., Lambert P.C., Rutherford M.J. (2020). Temporal Recalibration for Improving Prognostic Model Development and Risk Predictions in Settings Where Survival Is Improving over Time. Int. J. Epidemiol..

[24] Van Calster B., McLernon D.J., van Smeden M., Wynants L., Steyerberg E.W., Bossuyt P., Collins G.S., Macaskill P., McLernon D.J., Moons K.G.M. (2019). Calibration: The Achilles Heel of Predictive Analytics. BMC Med..

[25] Debray T.P.A., Vergouwe Y., Koffijberg H., Nieboer D., Steyerberg E.W., Moons K.G.M. (2015). A New Framework to Enhance the Interpretation of External Validation Studies of Clinical Prediction Models. J. Clin. Epidemiol..

[26] Janssen K.J.M., Moons K.G.M., Kalkman C.J., Grobbee D.E., Vergouwe Y. (2008). Updating Methods Improved the Performance of a Clinical Prediction Model in New Patients. J. Clin. Epidemiol..

[27] Riley R.D., Ensor J., Snell K.I.E., Debray T.P.A., Altman D.G., Moons K.G.M., Collins G.S. (2016). External Validation of Clinical Prediction Models Using Big Datasets from E-Health Records or IPD Meta-Analysis: Opportunities and Challenges. BMJ.

[28] Steif J., Brant R., Sreepada R.S., West N., Murthy S., Görges M. (2022). Prediction Model Performance With Different Imputation Strategies: A Simulation Study Using a North American ICU Registry. Pediatr. Crit. Care Med..

